# Self-awareness and Social Influences as Predictors of Body Dissatisfaction and Acceptance of Cosmetic Surgery for Social Reasons Among Men

**DOI:** 10.1007/s00266-023-03565-y

**Published:** 2023-08-28

**Authors:** Amanda Nerini, Cristian Di Gesto, Martina Lo Bartolo, Alessandro Innocenti, Cristina Stefanile, Camilla Matera

**Affiliations:** 1 Department of Education, Languages, Intercultures, Literatures and Psychology, University of Florence, Via di San Salvi, 12–Pad. 26, 50135 Florence, Italy; 2 School of Psychology, University of Florence, 50137 Florence, Italy; 3 Plastic and Reconstructive Microsurgery, Careggi University Hospital, Florence, Italy; 4 Department of Health Sciences, Section of Psychology, University of Florence, Via San Salvi 12, Pad. 26, Florence, Italy

**Keywords:** Self-awareness, Social influences, Body dissatisfaction, Acceptance of cosmetic surgery, Social reasons, Men

## Abstract

**Background:**

Body dissatisfaction and the use of surgery for purely aesthetic reasons among men is steadily increasing. Nevertheless, compared to women, few studies have focused on specific sociocultural and individual factors predicting men’s body dissatisfaction and interest in cosmetic surgery procedures. The present study investigated the role of media, significant others, public and private self-awareness in predicting men’s body dissatisfaction and acceptance of cosmetic surgery for social reasons.

**Methods:**

Participants were 203 men (mean-age 24 years), who completed a questionnaire containing the Sociocultural Attitudes Toward Appearance Questionnaire-4R, the Situational Self-Awareness Scale, the Muscular internalization subscale, the Male Body Attitudes Scale, and the Social subscale of the Acceptance of Cosmetic Surgery Scale. A path analysis was performed.

**Results:**

The influence of significant others and public self-awareness predicted men’s body dissatisfaction directly and indirectly, via muscularity internalization, while media was only directly associated with body dissatisfaction. A significant link between private self-awareness and body dissatisfaction was found. Moreover, media was not associated with cosmetic surgery either directly or indirectly and public self-awareness showed only a significant association with internalization.

**Conclusions:**

These findings provide information about the role that self-awareness and sociocultural factor play on body dissatisfaction and acceptance of surgery for social reasons among men. The study highlighted the importance of designing preventive programs aimed at enhancing men’s ability to resist various forms of pressure regarding body image and its management. Moreover, the advantages of focusing one’s attention on internal states and feelings can limit body dissatisfaction and can discourage consideration of cosmetic surgery for social benefits.

**Level of Evidence V:**

This journal requires that authors assign a level of evidence to each article. For a full description of these Evidence-Based Medicine ratings, please refer to the Table of Contents or the online Instructions to Authors www.springer.com/00266.

## Introduction

Physical appearance is increasingly important in Western societies [1]. Compared to women, relatively little attention has been paid to sociocultural and individual factors predicting men’s body dissatisfaction [2]. Although women are generally more dissatisfied with their bodies, men’s body dissatisfaction is steadily increasing and has been linked to negative outcomes, such as steroid use, muscle dysmorphia, eating disorders, low self-esteem, and substance abuse [3–5].

Social pressure to look good, transmitted and reinforced through social media, motivates the use of strategies for managing appearance, including surgery [6]. The normalization of physical appearance-enhancing behaviors has increased among men worldwide [7]. Cosmetic surgery interventions on men have increased by 335% from 1997 to 2019 [8]. In Italy, 16.9% of total cosmetic surgery in 2020 was performed on men, following international trends [9]. The age at which cosmetic surgery is sought has also decreased [10], with the 19–35 age group having the highest demand for cosmetic surgery among men. Many young men consider cosmetic surgery to improve love dating, marital satisfaction, job prospects, and to satisfy partners or meet stereotypical beauty ideals [11, 12]. However, accepting surgery for social benefits can lead to weaker psychological outcomes in terms of satisfaction [13].

Various types of cosmetic surgery are associated with several psychological risks in women and men, including, anxious and depressive symptoms, psychotic episodes, and post-traumatic stress disorder [14]. Postoperative complications of cosmetic procedures include neurological damage, brain damage, burns, chronic pain, necrosis, and, in extreme cases, death [15]. Body dissatisfaction following cosmetic surgery is also common among young women and men [16].

This evidence suggests that it is increasingly important to identify factors that can predict interest in undergoing cosmetic surgical procedures to modify their appearance in order to obtain social benefits not only among women, but also among men. Based on these considerations, this study aimed to identify sociocultural and individual factors predicting negative body image and acceptance of cosmetic surgery for social reasons among young men.

### The Role of Mass Media and Significant Others on Body Image and Cosmetic Surgery

The tripartite influence model (TIM) on body image [17, 18] suggests that appearance pressures from sociocultural agents (i.e., mass media, family, and peers) can influence body dissatisfaction and interest in cosmetic surgery among young men both directly and indirectly [19] fostering a greater tendency to incorporate idealized sociocultural aesthetic canons which emphasize strength and muscularity [20, 21]. As this internalization is often unfavorable in terms of self-evaluation [21], it results in increased body dissatisfaction and in a greater engagement in strategies aimed to modify one’s appearance (i.e., aesthetic surgery) [22]. Many studies [23, 24] confirmed that exposure to images of unrealistic idealized male bodies can lead to a greater internalization of muscular beauty ideals and contributes to body dissatisfaction and consideration of cosmetic surgery [25, 26]. Also significant others could reinforce compliance with physical muscularity standards. Correlational studies have shown that the internalization of these sociocultural messages may increase body concerns and the likelihood of considering body enhancement strategies [26, 27]. Men who perceive that their peers place high value on physical appearance for the purpose of gaining popularity, and who frequently discuss appearance-related topics with friends, are more inclined to consider cosmetic surgery for social reasons [26].

This study aims to investigate the role of both mass media and significant others in predicting negative body image and acceptance of cosmetic surgery for social motives among young men. To consider these sources of influence together, which has rarely been done so far, allows for a better understanding of the multiple factors influencing men’s appearance and body modification strategies.

### The Role of Self-Awareness on Body Image and Cosmetic Surgery

Although the TIM [17, 18] is strongly supported from an empirical point of view, some authors [28] have highlighted some limitations of the model, including the lack of individual variables that could contribute to a greater understanding of the etiology of negative body image. Tiggemann suggests that self-awareness is a relevant variable that can contribute to the formation of self-schemas related to physical appearance [28]. Self-awareness has two mutually exclusive dimensions: private, which involves attentiveness to the inner self, and public, which implies attentiveness to overt features of oneself and how one appears to others. Although self-awareness is an individual variable, its public dimension concerns a social area as it implies awareness of being the object of public observation and how one appears in the eyes of others [29].

Some studies [30, 31] have shown that self-awareness is relevant for body image and cosmetic surgery. Men with public self-awareness experience appearance-based worries and negative emotional states, leading to body dissatisfaction as the awareness of sociocultural beauty ideals increases. Moreover, public self-awareness is positively related to women’s acceptance of cosmetic surgery, as focusing on physical appearance increases motivation to meet social expectations and consider cosmetic procedures [31].

With regard to the link between private self-awareness and body image, only little empirical evidence has been provided so far. In a sample of male and female university students, Deinema [32] found that a higher focus on internal states led to more state appearance comparison among women, but not among male participants. Anyway, the authors did not explore the relationship between self-awareness and body dissatisfaction. Matera et al. [31] found that private self-awareness had a direct and negative relationship with acceptance of breast cosmetic surgery among women, suggesting that when individuals focus on inner aspects of the self (i.e., their thoughts and feelings), they are less likely to view cosmetic surgery positively.

To the best of our knowledge, no previous study has jointly considered the role of public and private self-awareness in predicting either body dissatisfaction or acceptance of cosmetic surgery among young men. Individuals aim to influence how others perceive them to achieve specific goals, such as encouraging observers to see them in a certain way, maintaining harmonious interactions to sustain relationships, or maximizing the social and material benefits of their interactions. To accomplish this, people tend to manage the impression they make on others by becoming highly self-aware and adapting their thoughts, emotions, and behaviors to meet others’ expectations and gain social recognition [33]. Considering that the tendency to focus on managing self-presentation is growing among men [34], it is reasonable to hypothesize a relevant role of self-awareness on both body dissatisfaction and acceptance of cosmetic surgery for them.

### The Present Study

The present study aimed to investigate the combined role of mass media, significant others, and both public and private self-awareness in predicting young men’s body dissatisfaction and acceptance of cosmetic surgery for social reasons. Although they are overall few, most of the studies [35, 36] that have investigated the use of cosmetic surgery among young men have involved university students. To obtain comparable findings, we aimed to test our hypotheses on a similar sample of young men.

Two statistical models were tested. In the first one, body dissatisfaction was the criterion variable. We hypothesized that significant others and media influences, together with public self-awareness, would be positively related to body dissatisfaction both directly and indirectly, via muscularity internalization. By increasing the salience that aesthetic standards have in the evaluation of one’s global self, the influence of both significant others and the media on body image, together with individuals’ awareness of how they appear publicly, would be associated with higher internalization of beauty ideals which, in turn, would predict greater body dissatisfaction (*Hypothesis 1a*). With respect to private self-awareness, we predicted a direct and negative link with men’s body dissatisfaction. Since private self-awareness implies a state of consciousness of one’s inner feelings, thoughts, and memories, it makes social factors (e.g., sociocultural standards of beauty) less accessible [37]. The more individuals focus on their internal states, the less they might perceive sociocultural pressures, thus experiencing lower body dissatisfaction (*Hypothesis 1b*).

In the second model, the acceptance of cosmetic surgery for social reasons was the criterion variable. Again, we hypothesized that the influence of media and significant others, together with public self-awareness, would be positively associated with the criterion variable both directly and indirectly, via muscularity internalization (*Hypothesis 2a*). Significant others and media can reinforce socially accepted men’s aesthetic standards; together with these sociocultural sources of influence, a focus on how individuals appear to others could favor the process of internalization, thus predicting higher acceptance of cosmetic surgery in order to adhere to the expectations of others and to obtain social benefits (e.g., being accepted). Differently, to focus one’s attention on internal states (i.e., private self-awareness) was hypothesized to be negatively associated with acceptance of cosmetic surgery for social reasons. Private awareness could weaken the salience of social body norms, thus reducing the interest in cosmetic procedures for obtaining social benefits (*Hypothesis 2b*).

## Method

### Participants

Participants were 203 men aged 18-35 years (*M* = 24.2, *DS* = 3.22). The mean body mass index (BMI) of the sample ranged from a minimum of 16.98 to a maximum of 31.35, with an average of 22.79 (*DS* = 2.77). All participants had Italian nationality. Most of them (63.1%) lived in central Italy, 31.5% in northern Italy and 5.4% in southern Italy and the islands.

Most of the participants declared to be unmarried (87.2%), 12.8% married or cohabiting. With regard to education, 77.8% of the participants had high school diplomas, 10.3% had bachelor’s degrees, 9.9% had completed middle school and 2% had master’s degrees. Concerning employment, 78.3% of the participants defined themselves as students, 21.7% defined themselves as workers (18.7% employed full time, 1.5% employed occasionally, 1% employed part time, and 0.5% unemployed).

### Measures

#### Sociocultural Influence

The influence of media and significant others on body image was assessed using, respectively, the *Pressures Media* subscale and *Significant Others* subscale of the Italian version [38] of the *Sociocultural Attitudes Toward Appearance Questionnaire*-4R (SATAQ-4R) [39]. The *Significant Others* subscale consists of five items (e.g., “I feel pressure from significant others to improve my appearance”; alpha = 0.91) rated on a 5-point Likert scale (1 = strongly disagree to 5 = strongly agree). Higher scores indicate higher levels of significant others’ influence. The *Pressures Media* subscale consists of six items (e.g., “I feel pressure from the media to improve my appearance”; alpha = 0.97) rated on a 5-point Likert scale (1 = strongly disagree to 5 = strongly agree). Higher scores indicate higher levels of media influence.

#### Self-awareness

In line with a previous study conducted with young Italian [31], public and private self-awareness was measured by some items taken from the *Situational Self-Awareness Scale* (SSAS) [40], of which three items related to private self-awareness (i.e., “Right now, I am conscious of my inner feeling”; “Right now, I am reflective about my life”) and three item related to public self-awareness (i.e., “Right now, I am concerned about the way I present myself”; “Right now, I am self-conscious about the way I look”). Items are rated on a 5-point Likert scale (1= totally disagree to 5= totally agree). Higher scores indicate higher levels of public and private self-awareness. Cronbach’s alphas were 0.88 and 0.90, respectively.

#### Internalization of Body Ideals

We used the Italian version of the *Muscular internalization* subscale [38] of the SATAQ-4R [39] to measure the internalization of body ideals. The subscale is composed of eight items (e.g., “It is important for me to look muscular”) rated on a 5-point Likert scale (*strongly disagree*) to 5 (*strongly agree*). Higher scores indicate higher levels of internalization of body ideals. Cronbach’s alpha was 0.90.

#### Male Body Dissatisfaction

The Italian version [41] of the *Male Body Attitudes Scale* (MBAS) [42] was used to assess body dissatisfaction in men. The purpose of the MBAS is to assess men’s attitudes toward their height, weight, and muscularity. The scale consists of twenty-seven items (e.g., “I think I have too little muscle on my body”; “I feel dissatisfied with my overall body build”). Each item is rated on a 6-point Likert scale (1=never to 6=always). Higher scores indicate higher levels of body dissatisfaction (alpha = 0.93).

#### Acceptance of Cosmetic Surgery Per Social Reasons

Acceptance of cosmetic surgery for social reasons was measured through the *Social* subscale of the Italian version [43] of the *Acceptance of Cosmetic Surgery Scale* (ACSS) [44]. This subscale assesses social motivations for having cosmetic surgery through 5 items (e.g., “If a simple cosmetic surgery procedure would make me more attractive to others, I would think about trying it”). It ranges from 1 (definitively disagree) to 7 (definitively agree). Higher scores indicate higher levels of acceptance of cosmetic surgery for social reasons (alpha = 0.94).

#### Sociodemographic Details, BMI, and Previous Cosmetic Surgery Interventions

Each participant reported his age, sex, nationality, educational level, occupational status, and relationship status. BMI (kg/m^2^) was calculated by asking participants to indicate their weight and height. Finally, the participants were asked if they had undergone cosmetic surgery interventions in the past.

### Procedure

Using opportunistic sampling techniques, participants were recruited from various places of aggregation, such as study rooms and libraries. We asked the participants to take part in a study on their own body opinions and habits. Participation was voluntary, and no incentives were provided. To be eligible for the study, participants had to be men at least 18 years of age. We obtained informed consent from each participant prior to administering the questionnaire.

Participants were asked to fill out a paper-and-pencil questionnaire anonymously and did not ask for any personally identifiable information. The questionnaire took about 20 minutes to complete. Administration took place in the presence of a researcher to whom participants could ask questions. The Ethical Committee of the University we are affiliated with approved the study procedures (Ref. No. 0174350/119).

### Data analyses

First, descriptive statistics (means and standard deviation) and intercorrelations between all the variables were performed. Second, we examined the fit of the two hypothesized models in which significant others, media influence, public and private self-awareness were posited, respectively, as predictors of men’s body dissatisfaction (Model 1) and acceptance of cosmetic surgery for social reasons (Model 2); the mediational role of muscularity internalization was considered in each model.

Less than 1% of the data was missing. We used a mean imputation process to replace the missing values. All the assumptions for path analysis were satisfied [45]. The hypotheses were tested using Amos (version 22); we used bootstrapping procedure [46] to test mediation by estimating the presence and size of the indirect (i.e., mediated) effects [47].

We adopted the maximum likelihood procedure to derive the parameter estimates and used the following goodness-of-fit indices: the *χ*^*2*^/df ratio, a good score of which is 2 or below; the comparative fit index (CFI); the Tucker–Lewis index (TLI); the incremental fit index (IFI), the value of which should be higher than 0.95; the normed fit index (NFI), a good score of which is more than 0.90; the root-mean-square error of approximation (RMSEA); a 90% confidence interval for RMSEA (RMSEA 90% CI); and the standardized root-mean-square residual (SRMR). RMSEA and SRMR are considered acceptable if they are 0.08 or lower [48].

## Results

None of the participants reported having undergone cosmetic surgery previously.

Table 1 shows the descriptive statistics (means and standard deviations) and the intercorrelations between all variables. No significant intercorrelations emerged between BMI, age, body dissatisfaction and acceptance of cosmetic surgery for social reasons. Significant others, media, public self-awareness, and internalization were positively associated with body dissatisfaction, while private self-awareness showed a negative link. Moreover, significant others and internalization were positively associated with acceptance of cosmetic surgery for social reasons, and private self-awareness showed a negative link, while no significant association was found for media and public self-awareness.

**Table 1:** Intercorrelation between all variables, means (*M*), and standard deviations (*SD*)

Variable	1	2	3	4	5	6	7	8	9	M (SD)
1. Age	1									24.21 (3.22)
2. BMI	.46***	1								22.79 (2.77)
3. Significant Others	.01	−.04	1							1.76 (.77)
4. Media	.23**	.13	.16*	1						2.25 (1.10)
5. Public Self-Awareness	.05	.05	−.01	−.04	1					3.28 (.70)
6. Private Self-Awareness	.06	.05	−.06	.07	.35***	1				3.63 (.82)
7. Internalization	.02	.19**	.35***	.04	.27***	.05	1			2.85 (.97)
8. Body Dissatisfaction	.06	.10	.27***	.13*	.12*	−.22**	.27***	1		2.85 (.88)
9. Acceptance of Cosmetic Surgery – Social	.04	−.03	.18**	.06	.01	−.16*	.12*	.13*	1	1.70 (1.22)

*N = *203; * p<.05; **p<.01;***p<.001

The data were normally distributed (skewness < 1.51; kurtosis < 4.09), as the skews for all variables were lower than 2 and kurtosis is lower than 7 [49]. Table 2 shows path analysis’s indices. Covariances ranged between 0.11 (*p* < 0.01) and 0.28 (*p* < 0.001).

**Table 2:** Path analysis’s indices

Fit indices	*χ*^*2*^	*p*	*χ*^*2*^*/df*	RMSEA	CI (Lower)	CI (Upper)	SRMR	CFI	TLI	IFI	NFI
Model 1 Body Dissatisfaction	8.48	0.20	1.41	0.05	0.00	0.11	0.04	0.98	0.95	0.98	0.93
Model 2 Acceptance of Cosmetic Surgery – Social	8.48	0.20	1.41	0.05	0.00	0.11	0.04	0.97	0.92	0.97	0.91

**Fig. 1:**
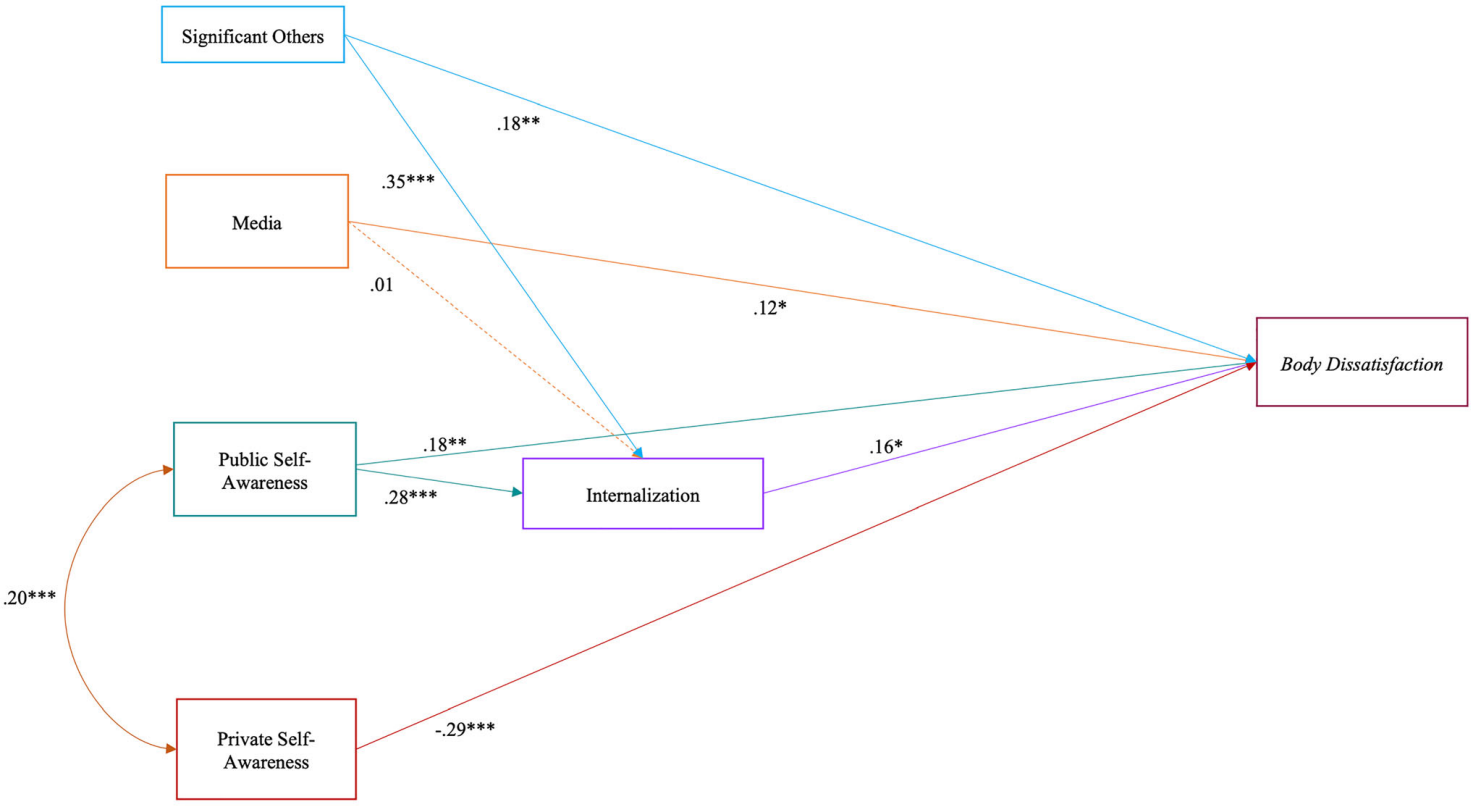
Model 1—Body dissatisfaction. *χ*^2^=8.48, *p* = 0.20; *χ*^2^/df=1.41; RMSEA=0.05 (CI=0.00; 0.11); SRMR=0.04; CFI=0.98; TLI=0.95; IFI=0.98; and NFI=0.93. *p<.05; **p<.01; and ***p<.001

Our hypotheses were partially confirmed. As for Model 1 (see Fig. 1), media showed a positive direct association with body dissatisfaction. Nevertheless, contrary to *Hypothesis 1a*, their effect was not mediated by muscularity internalization. In line with our first hypothesis, the influence of significant others and public self-awareness was significantly and positively associated with men’s body dissatisfaction both directly and indirectly, via muscularity internalization. Furthermore, in line with *Hypothesis 1b*, the association between private self-awareness and body dissatisfaction was negative and statistically significant.

**Fig. 2:**
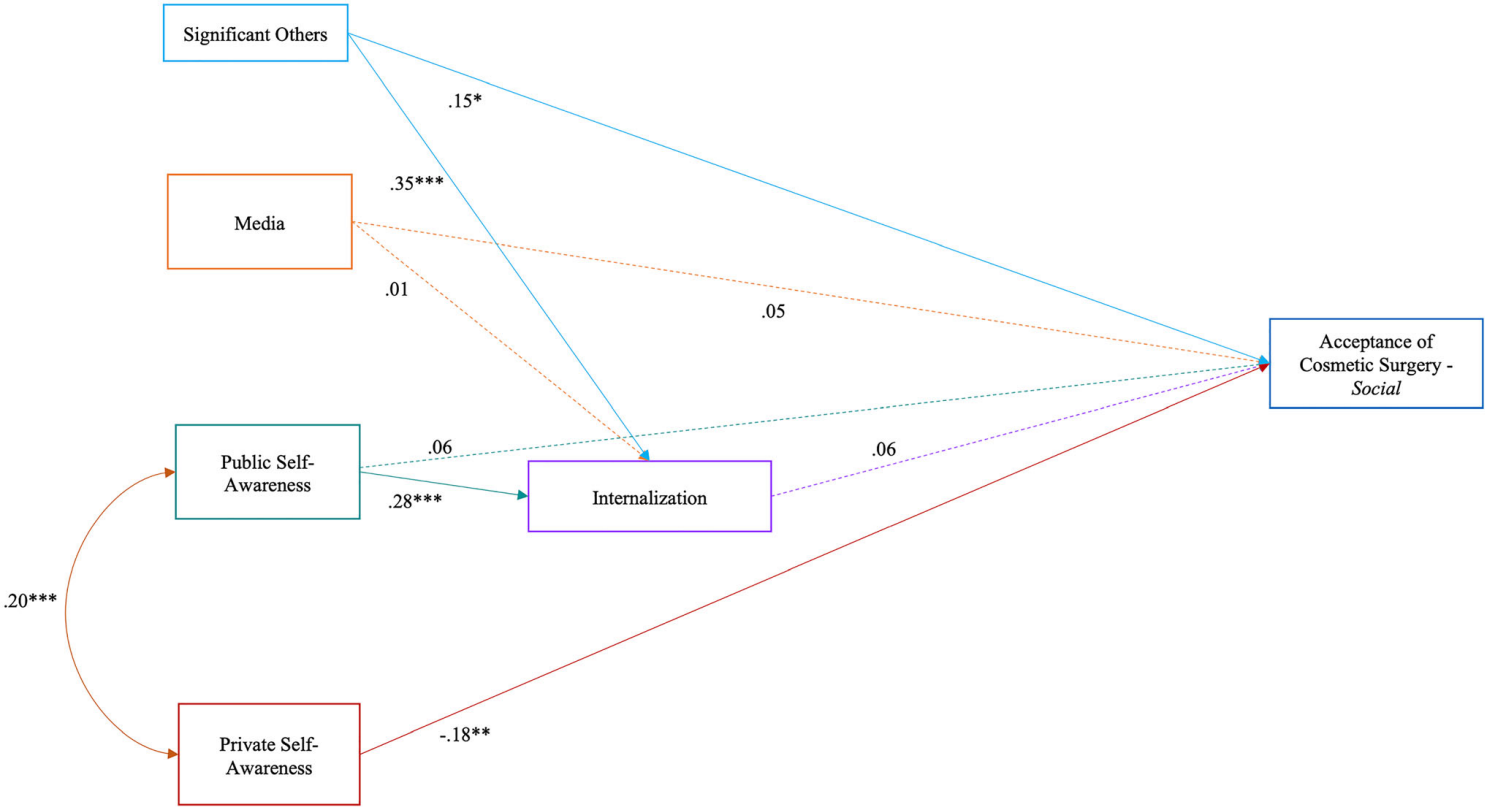
Model 2—Acceptance of cosmetic surgery—Social. *χ*^2^=8.48, *p* = 0.20; *χ*^2^/df=1.41; RMSEA=0.05 (CI=0.00; 0.11); SRMR=0.04; CFI=0.97; TLI=0.92; IFI=0.97; and NFI = 0.91 *p<.05; **p<.01; and ***p<.001

As for Model 2 (see Fig. 2), contrary to *Hypothesis 2a*, media influence was not associated with cosmetic surgery for social reasons either directly or indirectly. The influence of significant others was positively related to cosmetic surgery acceptance, while the indirect association was not significant, as we did not find an association between internalization and acceptance of aesthetic surgery. Contrary to our second hypothesis, public self-awareness was not linked to acceptance of cosmetic surgery for social reasons, although it was positively associated with internalization. Moreover, in line with *Hypothesis 2b*, a direct and negative association emerged between private self-awareness and acceptance of cosmetic surgery for social motivations.

Table 3 shows indirect effects values of path analysis.

**Table 3:** Indirect effects of path analysis

	Indirect effect	CI 95% (Lower)	CI 95% (Upper)
*Model 1*			
Media → Body dissatisfaction	0.900	−0.019	0.027
Significant others → Body dissatisfaction	0.017	0.012	0.112
Public self-awareness → Body dissatisfaction	0.010	0.010	0.099
*Model 2*			
Media → Acceptance of cosmetic surgery	0.950	−0.010	0.014
Significant others → Acceptance of cosmetic surgery	0.258	−0.018	0.058

Overall, the two statistical models accounted for a satisfactory percentage of the variance of body dissatisfaction (18%) and of acceptance of cosmetic surgery for social reasons (10%).

## Discussion

The aim of the present study was to examine the role of both sociocultural (i.e., significant others and media influence) and individual predictors (i.e., public and private self-awareness) of body dissatisfaction and acceptance of cosmetic surgery for social reasons among men.

Our findings partially supported our hypotheses concerning the influence of significant others and the media (*Hypotheses 1a* and *2a*). Significant others were found to be important sources of concern regarding physical appearance. This finding is in line with the scarce literature on men, showing that significant others can contribute to the onset of body dissatisfaction, especially when they foster a microculture focused on physical appearance [25, 50, 51]. These findings confirm that men’s bodies do not escape scrutiny by important others in their lives [52]. In line with the TIM [17, 18], our study showed that the influence of significant others on body image was partially mediated by the internalization of beauty ideals [53]. Also, the influence of significant others was found to be significantly associated with consideration of cosmetic surgery for social reasons. The study’s findings align with prior research indicating that exposure to significant others who promote unattainable beauty standards can lead young men to believe that these ideals reflect their actual appearance [19, 26]. Given the high desirability of these standards, the promotion of cosmetic surgery as a quick and accessible means of altering one’s body may make it a socially acceptable option. Contrary to *Hypothesis 2a*, the influence of significant others on conveying social norms regarding physical appearance did not act through muscular internalization.

A direct association between media influence and men’s body dissatisfaction emerged in our study, although the mediational role of the internalization of muscular beauty ideals was not confirmed. These findings suggest that young men are directly responsive to appearance-related signals conveyed by the media without filtering them through a preexisting set of internalized beauty standards. Media influence was not associated with cosmetic surgery for social reasons. This finding could be due to the assessment of media influence considering only traditional media and not social media. Indeed, previous studies [35, 54] showed that social rather than traditional media are a stronger predictor of acceptance of cosmetic surgery in men, as they portray these procedures as having minimal risks and significant social benefits [54]. Nevertheless, further research is needed to investigate the role that different media can have on men’s acceptance of cosmetic surgical procedures.

Our hypotheses concerning public (*Hypotheses 1b and 2b*) and private (*Hypotheses 1a and 2a*) self-awareness were partially confirmed. Public self-awareness was associated with body dissatisfaction via muscularity internalization, but not with acceptance of surgery for social reasons. This suggests that attentiveness to overt features of oneself, such as body appearance, and awareness of how one’s body appears publicly increase the influence of sociocultural standards of attractiveness, leading to their internalization. This is related to higher levels of body dissatisfaction, probably because the discrepancy between one’s real and ideal self is more accessible [55], but it may not be enough to motivate men to accept surgery for the purpose of gaining social benefits.

As regards *Hypotheses 1a and 2a*, we observed a negative and significant direct association between private self-awareness and both body dissatisfaction and acceptance of cosmetic surgery for social motivations. A state of private self-consciousness, characterized by higher awareness of one’s emotions, behaviors, values, and feelings [56], along with an increased salience of individual aspects to the detriment of sociocultural ones [57], may diminish the significance of prevailing aesthetic ideals in a sociocultural context, thus decreasing body dissatisfaction levels [58, 59]. Moreover, according to previous research on women [31], paying attention to internal aspects of oneself might make sociocultural pressures on body image less salient, thus reducing the likelihood of considering cosmetic surgery for social benefits.

This study has several strengths. To the best of our knowledge, it is one of the few investigating at the same time sociocultural and individual predictors of both body dissatisfaction and acceptance of cosmetic surgery for social reasons among men. Moreover, we analyzed the role of public and private self-awareness, which has been understudied, especially in the male population. Additionally, we focused on the social reasons that drive people to undergo cosmetic surgery, rather than looking at cosmetic surgery as a whole, which can allow for the development of more targeted prevention interventions.

Despite the strengths of our study, there are some limitations that should be acknowledged. First, the correlational research design does not allow for causal inferences to be made. Future longitudinal studies could shed light on the temporal relationship between the variables. Second, our sample consisted of Italian men aged 18–35, so the results may not be generalizable to other groups. Further studies could investigate the links between the variables in other age groups and cultural contexts where sociocultural pressures on body image are pervasive (e.g., adolescents, athletes). Third, we studied attitudes toward cosmetic surgery rather than actual use of surgery, so that we cannot establish if the factors identified through our study can also predict effective behavior. Although the statistical models we tested explained a satisfactory percentage of variance in body dissatisfaction levels (18%) and acceptance of cosmetic surgery for social reasons (10%), sociocultural and individual factors that can play a role in these variables are manifold. Future research should identify the role of additional factors, among which social media might have a pivotal role.

## Conclusions

In sum, our findings suggest that variables related to the social sphere, particularly the influence of significant others and the media, as well as being aware of how we can appear to others, predict greater body dissatisfaction among men; significant others also predict greater acceptance of cosmetic surgery. Conversely, when men focus on more intimate and private aspects, involving self-focused attention on internal cognitive and emotional states, they experience less body dissatisfaction and acceptance of surgery for obtaining social gains.

These findings have some potential implications. They could be useful in designing preventive programs directed to increase men’s ability to resist different forms of pressure, including that of significant others and media, on body image and its management. Some examples could include media literacy programs, and peer-led interventions aimed at instructing individuals on how to critically evaluate and analyze messages from media and significant others regarding body image.

Moreover, the advantages of focusing one’s attention on internal states and feelings were highlighted by our findings. Such a kind of attention can limit one’s body dissatisfaction and can discourage consideration of cosmetic surgery as an appearance modification strategy that can foster social benefits. Consequently, psychological approaches that encourage focusing on internal states (e.g., autogenic training, mindfulness, attention training technique, self-focusing attention training) might be especially useful for preventing dissatisfaction with one’s body image and related risky behaviors among men.
